# Proof-of-Concept Study of the NOTI Chelating Platform: Preclinical Evaluation of ^64^Cu-Labeled Mono- and Trimeric c(RGDfK) Conjugates

**DOI:** 10.1007/s11307-020-01530-8

**Published:** 2020-08-27

**Authors:** Sebastian Martin, Stephan Maus, Tobias Stemler, Florian Rosar, Fadi Khreish, Jason P. Holland, Samer Ezziddin, Mark D. Bartholomä

**Affiliations:** 1 Department of Nuclear Medicine, Saarland University – Medical Center, Kirrbergerstrasse, D-66421 Homburg, Germany; 2 Department of Nuclear Medicine and Molecular Imaging, Lausanne University Hospital, Rue de Bugnon 25A, CH-1011 Lausanne, Switzerland; 3 Department of Chemistry, University of Zurich, Winterthurerstrasse 190, CH-8057 Zurich, Switzerland; 4 Department of Nuclear Medicine, University of Freiburg – Medical Center, Hugstetterstrasse 55, 79106 Freiburg, Germany

**Keywords:** Copper-64, Integrins, α_v_ß_3_, PET imaging, Bifunctional chelator, Multimerization, Trimer, NODIA-Me, NOTI

## Abstract

**Purpose:**

We recently developed a chelating platform based on the macrocycle 1,4,7-triazacyclononane with up to three five-membered azaheterocyclic arms for the preparation of ^68^Ga- and ^64^Cu-based radiopharmaceuticals. Based on this platform, the chelator scaffold NOTI-TVA with three additional carboxylic acid groups for bioconjugation was synthesized and characterized. The primary aims of this proof-of-concept study were (1) to evaluate if trimeric radiotracers on the basis of the NOTI-TVA **6** scaffold can be developed, (2) to determine if the additional substituents for bioconjugation at the non-coordinating NH atoms of the imidazole residues of the building block NOTI influence the metal binding properties, and (3) what influence multiple targeting vectors have on the biological performance of the radiotracer. The cyclic RGDfK peptide that specifically binds to the α_v_ß_3_ integrin receptor was selected as the biological model system.

**Procedures:**

Two different synthetic routes for the preparation of NOTI-TVA **6** were explored. Three c(RGDfK) peptide residues were conjugated to the NOTI-TVA **6** building block by standard peptide chemistry providing the trimeric bioconjugate NOTI-TVA-c(RGDfK)_3_ **9**. Labeling of **9** with [^64^Cu]CuCl_2_ was performed manually at pH 8.2 at ambient temperature. Binding affinities of Cu-**8**, the Cu^2+^ complex of the previously described monomer NODIA-Me-c(RGDfK) **8**, and the trimer Cu-**9** to integrin α_v_ß_3_ were determined in competitive cell binding experiments in the U-87MG cell line. The pharmacokinetics of both ^64^Cu-labeled conjugates [^64^Cu]Cu-**8** and [^64^Cu]Cu-**9** were determined by small-animal PET imaging and *ex vivo* biodistribution studies in mice bearing U-87MG xenografts.

**Results:**

Depending on the synthetic route, NOTI-TVA **6** was obtained with an overall yield up to 58 %. The bioconjugate **9** was prepared in 41 % yield. Both conjugates [^64^Cu]Cu-**8** and [^64^Cu]Cu-**9** were radiolabeled quantitatively at ambient temperature in high molar activities of *A*_m_ ~ 20 MBq nmol^−1^ in less than 5 min. Competitive inhibitory constants IC_50_ of c(RDGfK) **7**, Cu-**8**, and Cu-**9** were determined to be 159.5 ± 1.3 nM, 256.1 ± 2.1 nM, and 99.5 ± 1.1 nM, respectively. In small-animal experiments, both radiotracers specifically delineated α_v_ß_3_ integrin-positive U-87MG tumors with low uptake in non-target organs and rapid blood clearance. The trimer [^64^Cu]Cu-**9** showed a ~ 2.5-fold higher tumor uptake compared with the monomer [^64^Cu]Cu-**8**.

**Conclusions:**

Functionalization of NOTI at the non-coordinating NH atoms of the imidazole residues for bioconjugation was straightforward and allowed the preparation of a homotrimeric RGD conjugate. After optimization of the synthesis, required building blocks to make NOTI-TVA **6** are now available on multi-gram scale. Modifications at the imidazole groups had no measurable impact on metal binding properties *in vitro* and *in vivo* suggesting that the NOTI scaffold is a promising candidate for the development of ^64^Cu-labeled multimeric/multifunctional radiotracers.

**Electronic supplementary material:**

The online version of this article (10.1007/s11307-020-01530-8) contains supplementary material, which is available to authorized users.

## Background

Bifunctional chelators (BFCs) are an integral part of metal-based, target-specific radiopharmaceuticals for cancer diagnosis and therapy. A BFC acts in two roles: (1) to stably complex the radiometal to prevent premature loss of the radiolabel *in vivo* and, thus, radioactivity accumulation in non-target tissues; and (2) to provide a second functionality for covalent linkage to a tumor-specific targeting vector. An ideal BFC should also be characterized by rapid complex kinetics and provide the radiotracer in high radiochemical yield (RCY) and high molar activity. In general, a metal-based target-specific radiotracer is comprised of one targeting vector coupled to one BFC. For the majority of commonly used macrocyclic BFCs such as DOTA (= 1,4,7,10-tetraazacyclododecane-1,4,7,10-tetraacetic acid) and NOTA (= 1,4,7-triazacyclononane-1,4,7-triacetic acid), one of the acetic acid arms is generally used for bioconjugation [1–4]. However, since the carboxylate-O donors of the acetic acid residues are also required for metal complexation, not more than one targeting moiety is usually attached to these BFCs *via* peptide bond formation to avoid the risk of compromising the metal binding, thus limiting their application to the design of monovalent radiotracers. Furthermore, other conjugation strategies such as click chemistry require additional modifications of these BFCs by either (1) coupling corresponding functionalities to the acetic acid residues through amide formation or (2) substitution of an acetic acid residue, thereby increasing the synthetic efforts [5–8]. Moreover, approaches for the design of multivalent/multimeric radiotracers usually connect multiple targeting vectors/biomolecules *via* additional aliphatic chains using, *e.g.*, poly-glycine, poly-lysine, poly-proline, or poly-ethyleneglycol linkers to which the metal chelator or ^18^F-radiolabeled prosthetic group is conjugated terminally [9–16]. This approach has commonly been used for an array of chelators including DOTA and NOTA, as well as for other radiolabels due to their limited number of functionalities for bioconjugation often only allowing the conjugation of a single biomolecule [9–16]. In addition to the additional synthetic steps, these aliphatic modifications/linkers however may increase the overall lipophilicity of the entire molecule, potentially resulting in unfavorable pharmacokinetics and high uptake in non-target organs.

**Scheme 1:**
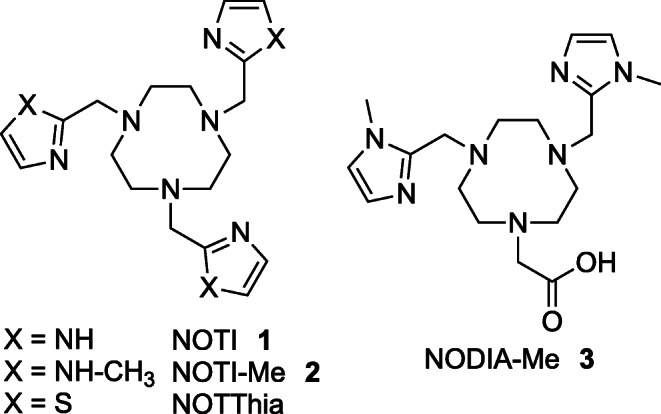
Chelating platform based on the TACN macrocycle with additional five-membered azaheterocyclic arms.

An alternative design of multivalent/multifunctional radiopharmaceuticals is the use of metal chelators that (1) intrinsically provide several functionalities for bioconjugation and (2) that can serve as the central unit of the radiotracer with no need for additional aliphatic linkages of the targeting vectors. In this respect, we recently introduced a chelating platform based on the macrocycle 1,4,7-triazacyclononane (TACN) with additional five-membered azaheterocyclic arms (Scheme 1) [17]. The chelators NOTI **1** (1,4,7-tris((1*H*-imidazol-2-yl)methyl)-1,4,7-triazonane), NOTI-Me **2** (1,4,7-tris((1-methyl-1*H*-imidazol-2-yl)methyl)-1,4,7-triazonane), and NOTThia (1,4,7-tris(thiazol-2-ylmethyl)-1,4,7-triazonane) were originally designed for the complexation of the positron-emitting radionuclide copper-64 which has favorable decay properties for PET (positron emission tomography). The half-life of ^64^Cu (*t*_1/2_ = 12.7 h) allows delayed imaging and investigation of biological processes at later time points for biomolecules/targeting vectors with intermediate pharmacokinetics such as peptides as well as nano- and minibodies. Before the availability of the positron-emitter ^89^Zr (*t*_1/2_ = 3.3 d), ^64^Cu has also been used for PET imaging with long-circulating biomolecules such as immunoglobulins or related fragments [18]. Moreover, the positron energy of ^64^Cu (*E*_β+,max_ = 653 keV; 18 %) is comparable with that of the PET gold standard ^18^F (*E*_β+,max_ = 633 keV) and its dual decay characteristics with its positron and beta (*E*_β-,max_ = 578 keV; 37 %) emissions allow diagnostic PET imaging and, in principle, radionuclide therapy with the same radiopharmaceutical [19–24]. Moreover, with its congener ^67^Cu (*t*_1/2_ = 61.5 h, *ß*^−^ 100 %, *E*_mean_ = 0.121 MeV), a therapeutic copper isotope is available allowing theranostic applications [25].

Recently, we showed that the series of TACN-based chelators NOTI **1**, NOTI-Me **2**, and NOTThia has excellent complexation properties for coordination of ^64^Cu^2+^ cations [17]. In extensive labeling experiments, these ligands demonstrated rapid complexation kinetics over a wide pH range of 4.0 to 8.0 providing quantitative radiochemical conversions under very mild conditions (< 5min incubation time, room temperature). Corresponding ^64^Cu complexes were obtained in high molar activities of 120–180 MBq nmol^−1^. In ligand challenge, copper exchange, and PET imaging experiments in mice, ^64^Cu complexes also demonstrated high kinetic inertness *in vitro* and *in vivo*. According to single crystal X-ray analyses of corresponding copper complexes, these excellent complexation properties are the result of the interplay between the optimal ring size of the TACN macrocycle and the pronounced affinity of divalent copper for the azaheterocyclic N donors. However, these ligands lacked a functionality that allowed their conjugation to target-specific biomolecules. As a consequence, the BFC NODIA-Me **3** (2-(4,7-bis((1-methyl-1*H*-imidazol-2-yl)methyl)-1,4,7-triazonan-1-yl)acetic acid), depicted in Scheme 1, was developed, in which one of the heterocyclic arms was replaced with an acetic acid group to serve as the site for the attachment of various targeting vectors. In subsequent studies, NODIA-Me **3** was successfully conjugated to a prostate-specific membrane antigen (PSMA) targeting vector [26]. In a more recent study using ^68^Ga- and ^64^Cu-labeled PSMA-targeting conjugates comprising NODIA-Me **3**, the applicability of this BFC in radiopharmaceutical development was successfully demonstrated [27]. Corresponding ^68^Ga- and ^64^Cu-labeled NODIA-Me-PSMA conjugates specifically delineated PSMA-positive LNCaP tumors with low background accumulation in small-animal PET imaging experiments and *ex vivo* biodistribution studies [27]. Noteworthy, no significant decomplexation/transchelation of the radiometal chelate was noted *in vitro* (< 2 % transchelation in copper exchange experiments over 24 h) and *in vivo* by PET imaging in healthy mice (rapid clearance with 1h post injection), underscoring the use of our chelating platform for radiopharmaceutical applications.

An intriguing finding of our previous studies was that modifications at the non-coordinating NH atoms of imidazole residues, *e.g.*, NOTI **1** *vs.* NOTI-Me **2**, did not alter the metal binding properties to a measurable extent [17]. This may offer the possibility of introducing additional functionalities to the metal chelator without compromising its complexation properties, potentially making the NOTI **1** scaffold a versatile tool for radiopharmaceutical development. To investigate this in more detail, we designed a trifunctional NOTI-TVA **6** building block with three additional carboxylic acid groups for the covalent conjugation of three targeting vectors *via* peptide bond formation. The primary aims of this proof-of-concept study were (1) to evaluate if trimeric radiotracers on the basis of the NOTI-TVA **6** scaffold can be developed, (2) to determine if the additional substituents for bioconjugation at the non-coordinating NH atoms of the imidazole residues of the building block NOTI influence the metal binding properties, and (3) what influence multiple targeting vectors have on the biological performance of the radiotracer. For this purpose, a trimeric c(RGDfK) conjugate was subsequently prepared on the basis of the NOTI-TVA scaffold **6**. Here, we report the ^64^Cu-labeling and preclinical evaluation of the trimeric c(RGDfK) bioconjugate *in vitro* and *in vivo* and provide a direct comparison to the monomeric counterpart. The c(RGDfK) targeting vector was chosen as a model for this proof-of-principle study since multiple multimeric radiotracers comprising two or more RGD targeting vectors have been thoroughly investigated due to the low expression of the corresponding α_v_ß_3_ integrin receptor. The results of this proof-of-concept study suggest that NOTI **1** is an ideal building block for the development of multifunctional radiotracers, considerably broadening the application scope of our chelating platform.

## Methods

### General

Chemicals and solvents were purchased from Sigma-Aldrich and TCI Europe, and used as received. The bifunctional chelator NODIA-Me **3** was prepared as previously described [28, 29]. The peptide c(RGDfK) **7** was purchased from ABX (Radeberg, Germany). The radioligand [^125^I]I-echistatin was obtained from Perkin Elmer (Boston, USA). High-resolution mass spectrometry ((+)-HR-ESI-MS) was performed on a Thermo Scientific Exactive mass spectrometer. [^64^Cu]CuCl_2_ was purchased from the Department of Preclinical Imaging and Radiopharmacy of the Eberhard-Karls-University (Tübingen, Germany). [^64^Cu]CuCl_2_ was produced *via* the ^64^Ni(p,n)^64^Cu route [30]. The corresponding molar activity was *A*_m_ = 300–400 MBq/nmol (18 h EOB). Proton NMR spectra were measured in MeOD or CDCl_3_ at room temperature using a Bruker DRX Avance 250 MHz NMR spectrometer. Chemical shifts are given in parts per million (ppm) and are reported relative to trimethylsilane (TMS). Coupling constants are reported in hertz (Hz). The multiplicity of the NMR signals is described as follows: s = singlet, d = duplet, t = triplet, q = quartet, m = multiplet. High-performance liquid chromatography (HPLC) was performed on an Agilent 1260 Infinity System equipped with an Agilent 1200 DAD UV detector (UV detection at 220 nm) and a Raytest Ramona radiation detector (Raytest GmbH, Straubenhardt, Germany) in series. A Phenomenex Jupiter Proteo (250 × 4.60 mm) column was used for analytical HPLC. The solvent system was A = H_2_O (0.1 % TFA) and B = acetonitrile (0.1 % TFA). The gradient was 0–1 min 5 % B, 1–25 min 50 % B, 25–27 min 95 % B, 27–29 min 95 % B, 29–32 min 5 % B, and 32–35 min 5 % B at a flow rate of 1 ml min^−1^. Semi-preparative HPLC was performed on a Knauer Smartline 1000 HPLC system in combination with a Macherey Nagel VP 250/21 Nucleosil 120-5 C_18_ column. Semi-preparative HPLC gradient was 0–40 min 5–60 % B at a flow rate of 12 ml min^−1^. For automated flash chromatography, a Biotage *Isolera Four* system was used. Normal phase chromatography was carried out by using a SNAP KP-Sil (50 g) column and n-hexane (D)/ethyl acetate (E) as eluents. The flow rate was 50 ml min^-1^. Detection wavelength was set at 280 nm. The normal phase gradient was 3 CVs (cartridge volumes) 0 % E, 3 CVs to 100 % E, and 4 CV 100 % E. Reverse phase flash chromatography was conducted with a SNAP Ultra C_18_ (30 g) cartridge. The solvent system was A = H_2_O and B = acetonitrile (both 0.1 % TFA). The flow rate was set at 25 ml min^−1^ with a detection wavelength of 280 nm. The reversed phase gradient was 2 CVs 0 % B, 6 CVs 100 % B, and 2 CVs 100 % B. Samples were lyophilized using a Christ Alpha 1-2 LD plus lyophilizer. All instruments measuring radioactivity were calibrated and maintained in accordance with previously reported routine quality control procedures [31]. Radioactivity was measured using an Activimeter ISOMED 2010 (Nuklear-Medizintechnik, Dresden, Germany). For accurate quantification of radioactivity, experimental samples were counted for 1 min on a calibrated Perkin Elmer (Waltham, MA, USA) 2480 Automatic Wizard Gamma Counter by using a dynamic energy window of 400–600 keV for copper-64 (511 keV emission). Statistical analyses (two-tailed Student’s *t* test, confidence interval 95 %) were performed using GraphPad Prism Version 7.0.

### Chelator and Bioconjugate Syntheses

#### Preparation of NOTI-TVA **6**

Route A: The NOTI **1** chelator was prepared as previously described in 17 % yield [17]. NOTI·3TFA (200 mg, 0.28 mmol) was reacted with methyl-5-bromovalerate (950 mg, 4.87 mmol) and cesium hydroxide hydrate (1.82 g, 10.8 mmol) in 25 ml of *N*,*N*-dimethylformamide (DMF) for 2 h at room temperature. After azeotropic removal of the DMF by co-evaporation with 5 × 50 ml toluene, the reaction mixture was acidified by addition of trifluoroacetic acid (TFA) until pH ~ 2 was achieved and further purified by reversed phase automated flash chromatography using C_18_ SNAP cartridges. The NOTI-TVA methyl ester **5** (280 mg, 0.27 mmol) was obtained as triple triflate salt in 65 % yield. Hydrolysis of methyl esters was achieved by heating compound **5** for 2 h at 95 °C in 10 ml of a H_2_O/TFA mixture (1:9, v/v) to give NOTI-TVA **6** (288 mg, 0.26 mmol) in quantitative yields.

#### Methyl 5-(2 formyl-1H-imidazol-1-yl)pentanoate **4**

Route B: Imidazole-2-carboxyaldehyde (3.0 g, 3.31 mmol), methyl-2-bromovalerate (5.34 ml, *ρ* = 1.363 g ml^−1^, 3.76 mmol), and potassium carbonate (8.62 g, 6.26 mmol) were mixed in 30 ml acetonitrile. The reaction was stirred for 24 h at 45 °C. Insoluble salts were filtered off and the brown liquid was concentrated by rotary evaporation to a volume of ~ 5 ml for the subsequent flash chromatography. Automated normal phase flash chromatography was applied for purification. The product **4** was obtained as colorless oil. Yield: 4.03 g, 19.1 mmol, 61 %. ^1^H-NMR (CDCl_3_): δ = 9.76 (s, 1H), 7.24 (d, *J* = 1.88 Hz, 1H), 7.14 (d, *J* = 1.9 Hz, 1H), 4.36 (t, *J* = 7.4 Hz, 2H), 3.62 (s, 3H), 2.31 (t, *J* = 7.0 Hz, 2H), 1.78 (m, 2H), 1.61 (m, 2H). HR-ESI-MS: calc. for C_10_H_15_N_2_O_3_ ([M + H]^+^): 211.1083, found: 211.1075. RP-HPLC (analytical): *t*_R_ = 9.2 min, purity > 99 %.

#### Trimethyl-5,5′,5″-(((1,4,7-triazonane-1,4,7-triyl)tris(methylene))tris(1H-imidazole-2,1-diyl))tripentanoate **5**

1,4,7-Triazacyclononane (200 mg, 1.55 mmol) and compound **4** (1.71 g, 7.74 mmol) were dissolved in 10 ml THF and the reactants heated at 70 °C for 24 h during the reaction solution became yellowish in color. After cooling to room temperature, sodium triacetoxyborohydride (2.0 g, 9.43 mmol) was added stepwise (300 mg every 30 min). After 5 h, 50 ml MeOH was added and the solvents were removed under reduced pressure generating a yellow oil. This was dissolved in 10 ml of a H_2_O/ACN (2:1, v/v) and the pH of the solution was carefully adjusted with TFA to pH 2–3. The crude mixture was purified by reversed phase automated flash chromatography. Fractions containing the product were combined and the solvents removed by rotary evaporation to give **5** as yellowish oil. Yield: 1.044 g, 1.47 mmol, 95 %. ^1^H-NMR (MeOD): *δ* = 7.61 (d, *J* = 1.44 Hz, 3H), 7.54 (d, *J* = 1.44 Hz, 3H), 4.37 (s, 6H), 4.18 (t, *J* = 5.86 Hz, 6H), 3.65 (s, 9H), 3.13 (s, 12H), 2.40 (t, *J* = 5.74 Hz, 6H), 1.82 (m, 6H), 1.61 (m, 6H). HR-ESI-MS: calc. for C_36_H_58_N_9_O_6_ ([M + H]^+^): 712.4505, found: 712.4506. RP-HPLC (analytical): *t*_R_ = 16.2 min, purity > 99 %.

#### 5,5′,5″-(((1,4,7-triazonane-1,4,7-triyl)tris(methylene))tris(1H-imidazole-2,1-diyl))tripentanoic acid (NOTI-TVA) **6**

For the hydrolysis of methyl esters, compound **5** (100 mg, 0.14 mmol) was heated at 95 °C for 24 h in 5 ml H_2_O/TFA (1:1, v/v). Solvents were removed under reduced pressure and the product lyophilized to obtain compound **6**. Yield: 93.6 mg, 0.14 mmol, quantitative. ^1^H NMR (MeOD): δ 7.61 (d, *J* = 1.68 Hz, 3H), 7.54 (d, *J* = 1.72 Hz, 3H), 4.42 (s, 6H), 4.23 (t, *J* = 7.3 Hz, 6H), 3.15 (s, 12H), 2.40 (t, *J* = 7.06 Hz, 6H), 1.90 (m, 6H), 1.64 (m, 6H). HR-ESI-MS: calc. for C_33_H_52_N_9_O_6_ ([M + H]^+^): 670.4035, found: 670.4042. RP-HPLC (analytical): *t*_R_ = 10.5 min, purity > 99 %.

#### NODIA-Me-c(RGDfK) **8**

**Scheme 2:**
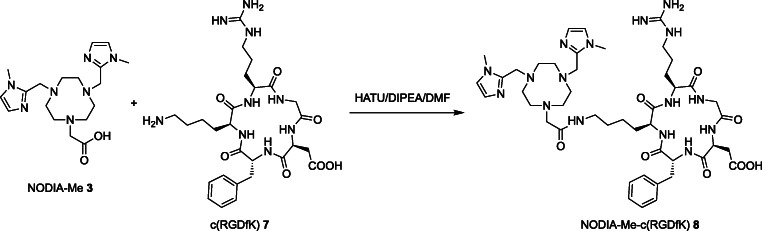
Two synthetic routes for the preparation of the trifunctionalized NOTI-TVA **6** scaffold.

The monomer **8** (Scheme 2) was prepared as previously described and characterization data was identical to that previously reported [29].

#### NOTI-TVA-c(RGDfK)_3_**9**

NOTI-TVA **6** (2 mg, 0.003 mmol), HATU **(**1-[bis(dimethylamino)methylene]-1*H*-1,2,3-triazolo[4,5-b]pyridinium-3-oxide hexafluorophosphate) (4 mg, 0.011 mmol), and DIPEA (*N*,*N*-diisopropylethylamine) (5.8 mg, 8 μl, 0.045 mmol) were mixed in 500 μl anhydrous DMF and allowed to stir for 30 min at room temperature. Next, the peptide c(RGDfK) **7** (6.3 mg, 0.009 mmol) in 100 μl DMF was added and stirring was continued for an additional 2 h. The solvent was removed by rotary evaporation and the residue was taken up in water/acetonitrile (50:50 v/v) with 0.1 % TFA and purified by semi-preparative HPLC. Fractions containing the product were combined and lyophilized to give compound **9** as white powder (3.0 mg, 0.001 mmol, 41 %). HR-ESI-MS calcd m/z for C_114_H_170_N_36_O_24_ ([M + 2H]^2+^): 1213.6578, found: 1213.6595. RP-HPLC (analytical): *t*_R_ = 16.5 min. RP-HPLC (semi-preparative): *t*_R_ = 18.0 min.

### Preparation of Copper Reference Compounds (Cu-8 and Cu-9)

From aqueous stock solutions of each bioconjugate (each 1 nmol μl^−1^), 500 μl were mixed with 500 μl of an aqueous CuCl_2_ stock solution (2 nmol μl^−1^) and the mixture heated at 95 °C for 15 min. After cooling to r.t., the complexes were purified using C_18_ Sep-Pak cartridges, which were preconditioned with each 5 mL of H_2_O and EtOH, respectively. After loading, the cartridges were washed with 2 ml H_2_O and the products were eluted using 2 ml EtOH:H_2_O (50:50 v/v). After evaporation of EtOH at ambient temperature, the remaining solutions were lyophilized to give Cu-**8** (450 μg, 0.44 μmol, 88 %) and Cu-**9** (1157 μg, 0.47 μmol, 93 %) as white powders. RP-HPLC (analytical) Cu-**8**: *t*_R_ = 15.7 min. HR-ESI-MS calcd m/z for C_45_H_68_N_16_O_8_Cu ([M]^2+^): 511.7351, found: 511.7348; RP-HPLC (analytical) Cu-**9**: *t*_R_ = 18.5 min. HR-ESI-MS calcd m/z for C_114_H_168_N_36_O_24_Cu ([M]^2+^): 1244.1164, found: 1244.1134.

### Radiolabeling of [^64^Cu]Cu-8 and [^64^Cu]Cu-9

Radiolabeling of **8** and **9** with [^64^Cu]CuCl_2_ was performed manually. In a typical labeling reaction, to solution of 450 μl, 0.5 M ammonium acetate (pH 8.2) was added 20 μl of the corresponding conjugate stock solution (1 nmol μl^−1^) followed by the addition of 200 MBq [^64^Cu]CuCl_2_ in 30 μl 0.1 M hydrochloric acid. Directly after the addition of the radioactivity, a sample of the labeling solution was analyzed by HPLC. The radiochemical purities (RCPs) and yields (RCYs) of both conjugates were always ≥ 99 %. The mean molar activities for both compounds were *A*_m_ ~ 20 MBq nmol^−1^ (each *n* = 5). RP-HPLC (analytical, radioactivity detector): *t*_R_ ([^64^Cu]Cu-**8**) = 16.3 min; *t*_R_ ([^64^Cu]Cu-**9**) = 18.7 min.

### Lipophilicity (Log D_oct/PBS_) Measurements

For log *D*_oct/PBS_ measurements, 1–2 MBq of [^64^Cu]Cu-**8** and [^64^Cu]Cu-**9** in 5 μl labeling buffer were added to a mixture of phosphate-buffered saline pH 7.4 (PBS) (495 μl) and octanol (500 μl). Samples were shaken for 30 min at room temperature, centrifuged at 13,200 rpm for 5 min, and 100 μl of each phase was counted using a gamma counter. Experiments were performed in triplicate.

### Serum Stability Measurements

For each experiment, ~ 5 MBq (10 μl) of [^64^Cu]Cu-**8** and [^64^Cu]Cu-**9** was added to human serum (490 μl, human male AB plasma, Sigma-Aldrich), which was pre-equilibrated at 37 °C in a cell incubator for 1 h. Samples were vortexed briefly and stored at 37 °C in a cell incubator. At selected time points, aliquots of 100 μl were taken from the serum solution and serum proteins removed by centrifugation using centrifuge tubes (molecular weight cutoff 30 kDa) at 4 G for 5 min at 4 °C. One hundred microliters of PBS were added to the filter followed by an additional centrifugation step. The corresponding filtrates were analyzed by HPLC. The percentage of intact [^64^Cu]Cu-**8** and [^64^Cu]Cu-**9** was calculated from the HPLC chromatograms. All experiments were performed in triplicate.

### Cell Culture

U-87MG cells (ATCC, Manassas, VA, USA) were cultured at 37 °C in a 5 % CO_2_ atmosphere (Dulbecco modified Eagle medium with GlutaMAX containing 10 % fetal bovine serum, 1 % 10,000 U ml^−1^ penicillin and 10,000 U ml^−1^ streptomycin, 1 % sodium-pyruvate 100 mM).

### Competitive Binding Assay

The binding affinity of Cu-**8** and Cu-**9** was determined by a cell-based competitive binding assay in the human glioma cell line U-87MG with [^125^I]I-echistatin as the radioligand as previously described [32]. Binding assays were performed in 24-well plates precoated with poly-l-lysine. Briefly, each compound at different concentrations (0–10,000 nM) was incubated for 2 h at r.t. with [^125^I]I-echistatin (30,000 cpm well^−1^) and 2 × 10^5^ U-87MG cells well^−1^. After incubation, cells were washed three times with ice-cold binding buffer and cell-associated activity recovered by addition of 1 M NaOH. Radioactivity was measured by a gamma counter and data fitted using non-linear regression (GraphPad Prism). Experiments were performed two times in triplicate.

### Cell Internalization Assay

For cell internalization, U-87MG cells were seeded at a density of 10^6^ cells per well in 6-well plates and incubated overnight with medium (Dulbecco modified Eagle medium with GlutaMAX containing 10 % fetal bovine serum, 1 % 10,000 U ml^−1^ penicillin and 10,000 U ml^−1^ streptomycin, 1 % sodium-pyruvate 100 mM). After 24 h, the medium was removed and the cells were washed with PBS. Approximately 2.5 pmol of [^64^Cu]Cu-**8** and [^64^Cu]Cu-**9** was added to the cells (in triplicates) to give a final volume of 1.5 ml PBS in each well followed by incubation for 1 h at 37 °C, 5 % CO_2_. To determine non-specific membrane binding and internalization, excess of c(RGDfK) (final concentration 1 μM) was added to selected wells. At each time point, the internalization was stopped by removing the medium and washing the cells twice with ice-cold PBS. To remove the receptor-bound radioligand, an acid wash was carried out twice with a 0.1 M glycine buffer pH 2.8 for 5 min on ice. Finally, cells were solubilized with 1 N NaOH. Radioactivity of cell fractions (supernatant, receptor-bound, and internalized) was measured by a Perkin Elmer gamma counter. Experiments were performed twice in triplicate.

### Small-Animal PET Imaging

All animal experiments complied with the current laws of the Federal Republic of Germany and were conducted according to German Animal welfare guidelines. Normal female athymic Balb/c nude mice (17–20 g, 4–6 weeks old) were obtained from Janvier SAS (St. Berthevin Cedex, France). Mice were provided with food and water *ad libitum*. U-87MG tumors were induced on the right shoulder by sub-cutaneous injection of 5 × 10^6^ cells in a 100 μl cell suspension of a 1:1 v/v mixture of media with reconstituted basement membrane (GFR BD Matrigel™, Corning BV, Amsterdam, Holland).

Static and dynamic PET imaging experiments were conducted on a microPET Focus 120 scanner (Concorde Microsystems) [33]. For dynamic PET imaging studies, approximately 5 min prior to recording PET images, mice (*n* = 3) were anesthetized by inhalation of 2–3 % isoflurane/oxygen gas mixture, fitted with an intravenous catheter and placed on the scanner bed in the prone position. Anesthesia was maintained using 1–2 % isoflurane. For all experiments, animals were injected with 100 μl sterile filtered phosphate-buffered saline formulations pH 7.4 of [^64^Cu]Cu-**8** and [^64^Cu]Cu-**9** (~ 500 pmol, 7–11 MBq). Dynamic scans were recorded from 0 to 1 h post radiotracer administration. List-mode data were acquired using a γ-ray energy window of 350–650 keV and a coincidence timing window of 6 ns. For the 24 h p.i. time point, mice were injected 24 h prior to image acquisition and placed in the cages. Five to ten minutes prior to static PET imaging, mice were anesthetized with isoflurane (2–4 % in air) and positioned in the scanner. Animals of the PET imaging studies were included in the *ex vivo* biodistribution studies (*vide infra*). Reconstruction was performed using unweighted OSEM2D. Image analysis was performed using AMIDE. Image counts per second per voxel (cps/voxel) were calibrated to activity concentrations (Bq ml^−1^) by measuring a 3.5-cm cylinder phantom filled with a known concentration of radioactivity. Specificity of [^64^Cu]Cu-**8** and [^64^Cu]Cu-**9** was confirmed by competitive inhibition (blocking) by co-injecting the peptide c(RGDfK) **7** (20 nmol mouse^−1^; *n* = 3).

### *Ex Vivo* Biodistribution

For each compound, a total of three animals were injected with [^64^Cu]Cu-**8** and [^64^Cu]Cu-**9** (~ 500 pmol, 7–11 MBq) in 100 μl sterile filtered phosphate-buffered saline *via* a tail vein. At 1 h p.i., animals were sacrificed by isoflurane anesthesia. Organs of interest were dissected, weighed, and assayed for radioactivity in a gamma counter. The percent injected activity per gram (%IA g^−1^) for each tissue was calculated by comparison of the tissue counts to a standard sample prepared from the injectate. Specificity of [^64^Cu]Cu-**8** and [^64^Cu]Cu-**9** was determined by co-injection of the peptide c(RGDfK) **7** (20 nmol mouse^−1^).

## Results

### Chelator and Bioconjugate Synthesis

**Scheme 3:**
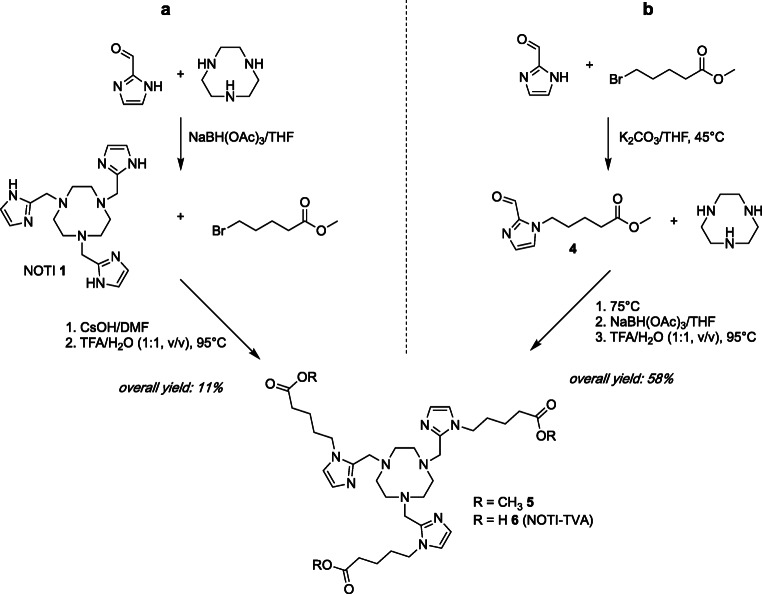
Synthetic scheme for monomeric bioconjugate NODIA-Me-c(RGDfK) **8**

To introduce additional functional groups to the NOTI **1** chelator for bioconjugation, two synthetic routes were explored (Scheme 3). Initially, the preparation of the trifunctionalized derivative started with the synthesis of NOTI **1** by reacting the 1,4,7-triazacyclononane (TACN) macrocycle with imidazole-2-carboxaldahyde and sodium borohydride in basic aqueous medium as previously described (route A) [17]. Under these reaction conditions, a mixture of di- and trisubstituted TACN was obtained providing the corresponding compounds di- and triderivatized compounds NODI and NOTI 1 in 46 % and 17 % yield, respectively. The NOTI **1** chelator then served as a building block for the preparation of the corresponding triply functionalized derivative NOTI-TVA **6**. The introduction of additional carboxylic acid functionalities for bioconjugation was achieved by reacting NOTI **1** with methyl-5-bromovalerate in the presence of cesium hydroxide in *N*,*N*-DMF at 60 °C for 2 h. This way, the triply derivatized NOTI-TVA (trivaleric acid) methyl ester **5** was obtained in 65 % yield. Subsequent hydrolysis of corresponding methyl esters in a H_2_O/TFA mixture at 95 °C for 2 h gave the NOTI-TVA **6** building block in 11 % overall yield.

In route B, the imidazole-2-carboxaldehyde was derivatized with methyl-5-bromovalerate prior to reacting with the TACN macrocycle. Reaction of the aldehyde with methyl-5-bromovalerate and potassium carbonate in *N*,*N*-DMF for 24 h at 45 °C (Scheme 3) proceeded smoothly and provided compound **4** in 61 % yield. NOTI-TVA methyl ester **5** was prepared by reductive amination in THF using sodium triacetoxyborohydride in 95 % yield. Noteworthy, under these conditions, only the triply derivatized NOTI-TVA methyl ester **5** was obtained. Removal of the methyl esters was achieved again with 5 M hydrochloric acid providing the NOTI-TVA **6** scaffold in 58 % overall yield.

**Scheme 4:**
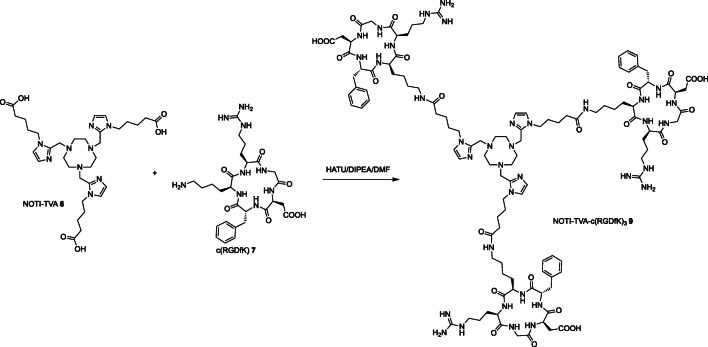
Preparation of trimeric bioconjugate NOTI-TVA-c(RGDfK)_3_ **9**.

The homotrimeric NOTI-TVA-c(RGDfK)_3_ bioconjugate **8** was prepared by reacting NOTI-TVA **6** with 3.5 equivalents of the commercially available c(RGDfK) peptide **7** in the presence of HATU (*O*-(7-azabenzotriazol-1-yl)-*N*,*N*,*N*′,*N*′-tetramethyluronium-hexafluorphosphat) and *N*,*N*-diisopropylethylamine (DIPEA) in DMF (Scheme 4). After purification by semi-preparative HPLC, the final bioconjugate NOTI-TVA-c(RGDfK)_3_ **9** was successfully obtained in 41 % yield. Corresponding characterization data of intermediates and bioconjugates including NMR, MS, and HPLC data are provided as Electronic Supplementary Material. For comparison reasons (*vide infra*), the monomeric compound NODIA-Me-c(RGDfK) **8** was prepared as previously reported and included in this study (Scheme 3) [29].

### ^64^Cu-Labeling and Characterization *In Vitro*

Both the monomer NODIA-Me-c(RGDfK) **8** and the trimer NOTI-TVA-c(RGDfK)_3_ **9** were labeled manually with [^64^Cu]CuCl_2_ in 0.5 M ammonium acetate (pH 8.2). Quantitative radiochemical yields were achieved for both conjugates at room temperature in less than 5 min of incubation time. Radiolabeling conditions were chosen to obtain both tracers in molar activities of ~ 20 MBq nmol^−1^. Corresponding radiochemical purities (RCP) were ≥ 99 % for both compounds. Both radiolabeled compounds gave a single radioactive peak in the analytical HPLC and their identity was confirmed using their non-radioactive reference counterparts (Figs. S10+11). In serum stability studies, no demetalation or other metabolites were detected up to 24 h by radio-HPLC. Corresponding log D_oct/PBS_ values of [^64^Cu]Cu-**8** and [^64^Cu]Cu-**9** were − 3.69 ± 0.15 and − 3.14 ± 0.12, respectively.

**Fig. 1.**
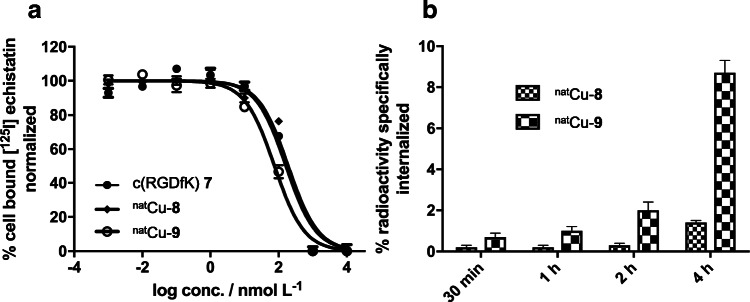
**a** Competitive inhibition of [^125^I]I-echistatin by c(RGDfK) **7**, Cu-**8** and Cu-**8** in the U-87MG cell line. **b** Receptor-mediated internalization of [^64^Cu]Cu-**8** and [^64^Cu]Cu-**9** into U-87MG cells over time.

The binding affinity of Cu-**8** and Cu-**9** to the α_v_ß_3_ integrin receptor was evaluated by competitive cell binding experiments in the α_v_ß_3_-positive U-87MG glioblastoma cell line. Both compounds inhibited the binding of [^125^I]I-echistatin in a dose-dependent manner (Fig. 1a) and corresponding IC_50_ values for c(RGDfK) **7**, Cu-**8**, and Cu-**9** were determined to be 159.5 ± 1.3 nM, 256.1 ± 2.1 nM, and 99.5 ± 1.1 nM, respectively. In addition to the inhibitory binding constants, the receptor-mediated internalization of [^64^Cu]Cu-**8** and [^64^Cu]Cu-**9** in U-87MG cells was also evaluated over a 4-h time period (Fig. 1b). Both compounds showed a gradually increasing receptor-mediated internalization over time. For the monomer [^64^Cu]Cu-**8**, an α_v_ß_3_-specific increase in internalization from 0.2 ± 0.1 % after 30 min to 1.4 ± 0.1 % at 4 h was noted, whereas the trimer [^64^Cu]Cu-**9** exhibited an increased internalization with 0.7 ± 0.2 % after 30 min and 8.7 ± 0.6 % after 4 h. In fact, the trimer displayed a significantly higher internalization than the monomer at later time points (2 h, *P* = 0.016; 4 h, *P* = 0.004) being in accordance with the results of the competitive binding experiment. On the other hand, the specific surface-bound fraction for [^64^Cu]Cu-**8** and [^64^Cu]Cu-**9** was low with ≤ 0.1 % at all time points.

### *Ex Vivo* Biodistribution and Small-Animal PET Imaging

**Fig. 2.**
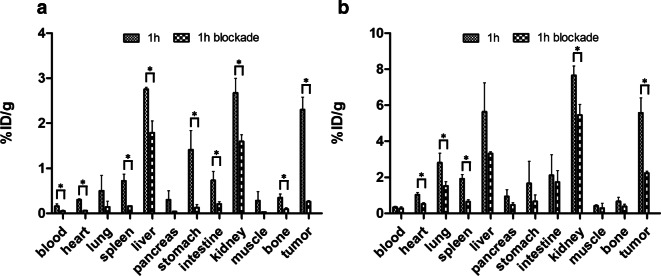
*Ex vivo* biodistribution data for [^64^Cu]Cu-**8** (**a**) and [^64^Cu]Cu-**9** (**b**) in female U-87MG xenograft bearing BALB/c mice at 1-h post-injection including blockade. Data given as percent injected activity per gram ± standard deviation (%IA g^−1^ ± SD). Statistical significance between the normal and blocking groups was determined by an unpaired two-tailed *t* test and is indicated by an asterisk (*P* < 0.05).

The biodistribution for [^64^Cu]Cu-**8** and [^64^Cu]Cu-**9** was assessed in female BALB/c mice bearing U-87MG xenografts. A graphical representation of the results of the *ex vivo* biodistribution study including blockade at 1 h post-injection (p.i.) are given in Fig. 2. Data is provided in tabular form as Electronic Supplementary Material (Table S1).

In accordance with the results of the cell experiments, the trimer [^64^Cu]Cu-**9** exhibited a significantly higher tumor uptake than the monomer [^64^Cu]Cu-**8** with 2.30 ± 0.28 %IA g^−1^ and 5.57 ± 0.83 %IA g^−1^ at 1 h p.i. (*P* = 0.003), respectively. Target specificity was confirmed by co-injection of c(RGDfK) **7**, which significantly reduced the uptake in U-87MG xenografts by 89 % (*P* = 0.0002) and 60 % (*P* = 0.002) for [^64^Cu]Cu-**8** and [^64^Cu]Cu-**9**, respectively. The highest tracer accumulation for [^64^Cu]Cu-**9** was observed in the liver with 5.63 ± 1.61 %IA g^−1^ and the kidneys with 7.65 ± 0.52 %IA g^−1^, suggesting excretion of the tracer *via* the hepatobiliary and renal pathway. Kidney and liver uptakes were blockable with 3.32 ± 0.09 %IA g^−1^ (*P* = 0.0084) and 5.45 ± 0.59 %IA g^−1^ (*P* = 0.1038), respectively, indicating that accumulation in both organs was also partially receptor mediated. Radiotracer accumulation in the blood at 1 h p.i. was low for both compounds with 0.16 ± 0.05 %IA g^−1^ for the monomer and 0.34 ± 0.05 %IA g^−1^ for the trimer, resulting in high tumor-to-blood ratios of 14.37 and 16.38, respectively.

**Fig. 3.**
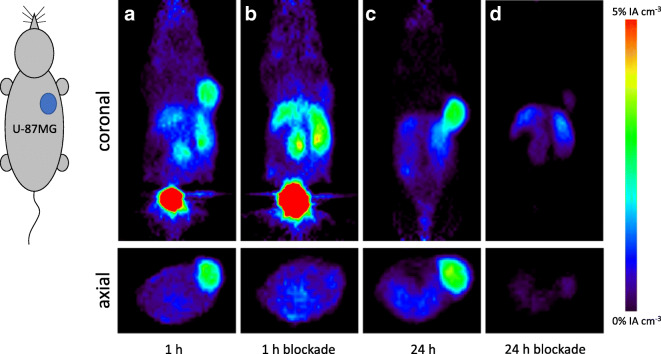
Representative transverse and coronal PET images of [^64^Cu]Cu-**8** in female U-87MG xenograft bearing BALB/c mice at 1 and 24 h (**a** + **c**) post-injection including blockade by co-injection of c(RGDfK) (**b** + **d**).

**Fig. 4.**
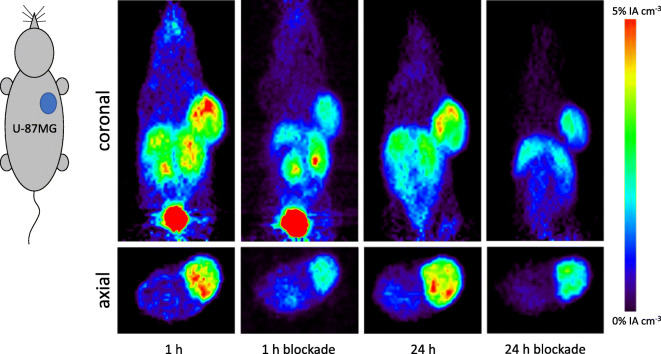
Representative transverse and coronal PET images of [^64^Cu]Cu-**9** in female U-87MG xenografts bearing BALB/c mice at 1 and 24 h (**a** + **c**) post-injection including blockade by co-injection of c(RGDfK) (**b** + **d**).

The pharmacokinetic profiles of ^64^Cu-labeled **8** and **9** were also determined by small-animal PET imaging. Dynamic PET imaging in BALB/c mice bearing U-87MG xenografts, along with blocking studies, was performed for 1 h after radiotracer administration. An additional static PET scan was performed at 24 h p.i. Corresponding transverse and coronal PET images of [^64^Cu]Cu-**8** and [^64^Cu]Cu-**9** are given in Figs. 3 and 4, respectively. Tumor uptakes of [^64^Cu]Cu-**8** and [^64^Cu]Cu-**9** at 1 h p.i. was 2.61 ± 0.65 %IA cm^−3^ and 4.42 ± 1.10 %IA cm^−3^, respectively. Target specificity was confirmed by co-injection of c(RGDfK) (5 mg/kg) resulting in a significant reduction of tumor accumulation with 0.91 ± 0.39 %IA cm^−3^ and 2.18 ± 0.66 %IA cm^−3^ for [^64^Cu]Cu-**8** and [^64^Cu]Cu-**9** as well as reduced uptake in all other organs.

## Discussion

For this proof-of-principle study, the cyclic RGDfK **7** peptide was selected as the targeting vector which binds specifically to the α_v_ß_3_ integrin receptor. The integrin α_v_ß_3_ receptor is upregulated in activated endothelial cells of tumors undergoing angiogenesis but is not expressed in normal cells and quiescent vessel cells making it a key target for the diagnosis of malignant tumors and metastases [34, 35]. Besides applications in oncology, imaging of α_v_ß_3_ expression is also applied in cardiology and inflammatory diseases [36, 37]. Due to the low expression profile of the α_v_ß_3_ integrin receptor, this peptide/receptor couple was one of the first systems for which multimeric/multivalent radiotracers were developed in order to increase the tumor uptake by making use of the multivalency effect [12, 13, 16, 38–40]. This seemed to be the appropriate biological system to evaluate the potential of our chelating platform since up to three targeting vectors can potentially be covalently conjugated to the NOTI-TVA scaffold **6** *via* peptide bond formation.

In order to establish a reliable synthetic route for the chelator scaffold, the two synthetic pathways for the preparation of the NOTI-TVA **6** were explored. The initial synthesis started with the recently described chelator NOTI **1** to which valeric acid residues were introduced *via* nucleophilic substitution. However, the overall yield of route A (11 %), depicted in Scheme 3, was quite low being a result of the low yielding synthesis of NOTI **1** due to the very limited solubility, and thus limited reactivity of the imidazole-2-carboxaldehyde in organic solvents and aqueous media [17]. On the other hand, the introduction of the valeric acid residues and the subsequent cleavage of corresponding methyl esters proceeded smoothly in quantitative yields. This straightforward and high yielding derivatization of imidazoles led to the exploration of an alternative synthetic route (route B) to obtain the NOTI-TVA **6** scaffold in higher overall yields (Scheme 3). Here, the imidazole carboxaldehyde was derivatized with valeric acid prior to reacting with the TACN macrocycle. The functionalization of the NH group of the imidazolecarboxaldehyde significantly increased its solubility and reactivity in organic solvents resulting in a fivefold improvement in overall yield (58 %) in comparison with route A (overall yield 11 %). This approach provides access to the NOTI-TVA **6** scaffold in high yield but, more importantly, also nicely demonstrates the potential and flexibility of the NOTI chelating platform. The possibility to straightforwardly derivatize the imidazole residues by nucleophilic substitution allows the development of various functionalized NOTI derivatives including other bioconjugation strategies in high yield for future studies. The corresponding NOTI-TVA-c(RGDfK)_3 _**9** conjugate for this proof-of-principle study was prepared by standard peptide chemistry demonstrating that homotrimeric compounds based on the NOTI-TVA **6** scaffold are readily accessible by this approach. In subsequent ^64^Cu-labeling experiments and serum stability studies, no significant differences in terms of radiolabeling and *in vitro* stability compared with the non-derivatized chelators were noted suggesting that the additional valeric acid substituents are well tolerated in terms of metal chelation [17].

Moreover, we were interested in the influence of the metal chelates on the lipophilicity of corresponding bioconjugates to elucidate if a central chelating unit is a feasible approach for the development of multimeric/multivalent radiotracers. As a measure of lipophilicity, corresponding partition coefficients log D_oct/PBS_ were determined for the ^64^Cu-labeled monomer and trimer. The ^64^Cu-labeled monomer **8** was only slightly more lipophilic than its ^68^Ga-labeled counterpart [^68^Ga]Ga-**8** with a log D_oct/PBS_ of − 3.89 ± 0.02 [29]. In line with the HPLC retention times, the ^64^Cu-labeled trimer **9** was only minimally more lipophilic than the monomer **8** despite its relatively high molecular weight in comparison with the monomer **8** (961 g mol^−1^*vs.* 2426 g mol^−1^). Noteworthy, the ^64^Cu-labeled trimer **9** was more hydrophilic than other monomeric RGD radiotracers such as [^64^Cu]Cu-CB-TE2A-c(RGDfK), [^64^Cu]Cu-NODAGA-c(RGDfK), and [^64^Cu]Cu-DOTA-c(RGDfK) with log D_oct/PBS_ of − 2.92, − 2.76, and − 2.77, respectively [32]. This may be a consequence of the high hydrophilicity of ^64^Cu chelates of NOTI **1**, NOTI-Me **2**, and NOTThia, making them ideal candidates for increasing the overall hydrophilicity of lipophilic targeting vectors [17]. The results also suggest that connecting multiple targeting vectors *via* a central metal binding moiety as it is the case for the NOTI-TVA scaffold **6** may be superior to other approaches in which multiple targeting vectors/biomolecules are connected *via* aliphatic linkers and, then, conjugated terminally to the metal chelator or ^18^F-radiolabeled prosthetic group. This approach has been commonly used for many metal chelators, *e.g.*, DOTA or NOTA, or other radiolabels due to their limited number of functionalities for bioconjugation often only allowing the conjugation of a single biomolecule [9–16].

To study the effect of multiple RGD targeting vectors on the target binding, the binding affinity of Cu-**8** and Cu-**9** to the α_v_ß_3_ integrin receptor was evaluated by competitive cell binding experiments in the α_v_ß_3_-positive U-87MG glioblastoma cell line. For the monomer Cu-**8**, the introduction of the NODIA-Me chelate to the peptide had only a minimal effect on receptor binding as already seen for the corresponding Ga-**8** monomer with an IC_50_ = 205.1 ± 1.4 nM [29]. Interestingly, the trimeric compound Cu-**9** displayed an increased binding affinity compared with the Ga-**8** and Cu-**8** monomers suggesting that multimerization of targeting vectors using the NOTI platform may result in higher binding affinity. Given the short distance between the c(RGDfK) peptide residues (∼ 20 bond distances) of the trimer, it is unlikely that they would bind simultaneously to adjacent α_v_ß_3_ integrin receptors to achieve a multivalency effect. However, the binding of one c(RGDfK) motif to the receptor significantly increases the “local concentration” in the vicinity of the receptor binding site. This might lead to an enhanced α_v_ß_3_ integrin binding rate or a reduced dissociation rate of the c(RGDfK) peptide from the α_v_ß_3_ integrin receptor. Even though a direct comparison to other studies is not possible due to differences in the experimental design, *e.g.*, different cell line, the determined IC_50_ values are in accordance with those of previously reported monomeric RGD radiotracers [32]. The receptor-mediated internalization of [^64^Cu]Cu-**8** and [^64^Cu]Cu-**9** was furthermore evaluated for up to 4 h. Cellular internalization upon receptor binding is believed to translate into prolonged tumor retention *in vivo*. Both radiotracers were specifically internalized with the [^64^Cu]Cu-**9** trimer exhibiting a significantly higher internalization than the monomer being in accordance with the results of the competitive binding experiment. In summary, a higher binding affinity as well as specific cellular internalization was noted for the trimer in comparison with its monomeric counterpart suggesting that the use of several targeting moieties based on the NOTI-TVA chelating platform may lead to superior tumor binding and accumulation.

To elucidate if the higher binding affinity and cellular internalization of the trimer translated to an increased tumor uptake *in vivo*, we assessed the biodistribution for [^64^Cu]Cu-**8** and [^64^Cu]Cu-**9** in female BALB/c mice bearing U-87MG xenografts. Indeed, the trimer [^64^Cu]Cu-**9** exhibited a significantly higher tumor uptake than the monomer [^64^Cu]Cu-**8** with 2.30 ± 0.28 %IA g^−1^ and 5.57 ± 0.83 %IA g^−1^ at 1 h p.i. (*P* = 0.003), respectively. Interestingly, the tumor uptake of the trimeric compound lies in between values reported for ^64^Cu-labeled dimeric and tetrameric RGD bioconjugates with ~ 4 and ~ 10 %IA g^−1^, respectively [11]. In comparison with the monomer, the trimer exhibited a higher tracer accumulation in all organs, except for the stomach and the muscles. The increased organ uptake may be explained by the higher number of RGD targeting vectors with a higher binding affinity and avidity. This is supported by the results of the blocking study, in which the uptake in normal organs was significantly reduced, confirming receptor mediated radiotracer accumulation also in normal tissues. This is in accordance with the literature, where α_v_ß_3_ imaging probes demonstrated low but blockable uptake in normal tissues [41–45]. However, the blocking effect was less pronounced for the trimer than for the monomer, which may be a result of the enhanced tumor binding due to the three RGD targeting vectors and/or a slightly higher fraction of non-specific binding. An amount of 5 mg/kg c(RGDfK) was injected in the blocking group, which has previously been used for confirming target specificity but this amount may have been too low to achieve a more pronounced blocking effect for the trimer [32]. The highest radioactivity accumulation for [^64^Cu]Cu-**9** was observed in the liver with 5.63 ± 1.61 %IA g^−1^ and the kidneys with 7.65 ± 0.52 %IA g^−1^, suggesting excretion of the tracer *via* the hepatobiliary and renal pathway. Kidney and liver uptakes were blockable with 3.32 ± 0.09 %IA g^−1^ (*P* = 0.0084) and 5.45 ± 0.59 %IA g^−1^ (*P* = 0.1038), respectively, indicating that accumulation in both organs was also partially receptor mediated. Reported values for both organs are comparable with the liver and kidney uptakes of a ^64^Cu-labeled tetrameric c(RGDfK) bioconjugate [11]. The kidney and liver uptakes for the monomer [^64^Cu]Cu-**8** were 2.67 ± 0.33 %IA g^−1^ and 2.75 ± 0.05 %IA g^−1^, respectively, comparable with those of other ^64^Cu-labeled RGD monomeric radiotracers [32, 46]. For both tracers, the tumor-to-liver and tumor-to-kidney ratios of 0.84 and 0.86 for the monomer and 0.99 and 0.73 for the trimer were moreover in accordance with ratios of recently reported ^64^Cu-labeled RGD monomeric analogs ranging from 0.70 to 1.13 [32, 46]. In that series, RGD radiotracers comprised different metal chelators including NOTA-Bn-NCS and several TE2A derivatives including cross-bridged TE2A [32, 46]. Despite the relatively high molar mass of the trimer, the blood uptake at 1 h p.i. was comparable or even lower than that of other ^64^Cu-labeled radiotracers bearing only a single RGD targeting vector such as DOTA-c(RGDfK), NODAGA-c(RGDfK), and CB-TE2A-c(RGDfK) with 0.63 ± 0.14 %IA g^−1^, 0.31 ± 0.09 %IA g^−1^, and 0.49 ± 0.05 %IA g^−1^, respectively [32].

**Fig. 5.**
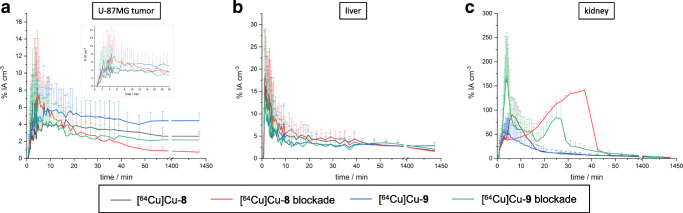
Representative time-activity curves (TACs) of [^64^Cu]Cu-**8** and [^64^Cu]Cu-**9** for the U-87MG tumor (**a**) with inlet for 0–20 min p.i., liver (**b**), and kidneys (**c**). Data given as percent injected activity per cubic centimeter ± standard deviation (%IA cm^−3^ ± SD).

In addition to the *ex vivo* biodistribution studies, the pharmacokinetics of [^64^Cu]Cu-**8** and [^64^Cu]Cu-**9** were determined by small-animal PET imaging in mice bearing U-87MG xenografts. As can be seen in Figs. 3 and 4, respectively, the α_v_ß_3_-positive U-87MG tumors as well as the liver and the kidneys were clearly visualized by [^64^Cu]Cu-**8** and [^64^Cu]Cu-**9** on the PET images confirming the results of the biodistribution study with the trimer [^64^Cu]Cu-**9** exhibiting a ~ 2-fold higher tumor uptake compared with its corresponding monomeric part. As illustrated by corresponding time-activity curves (TACs) in Fig. 5 obtained from dynamic PET imaging from 0 to 1 h p.i., tumor accumulation for [^64^Cu]Cu-**8** and [^64^Cu]Cu-**9** was rapid with both compounds reaching maximum after ~ 5–10-min post-administration followed by a decline until 20 min p.i. with no significant differences between the non-blocking and blocking groups. After 20 min p.i., tumor uptake for both radiotracers stabilized and persistent retention was noted up to 24 h for both radiotracers with 2.80 ± 0.52 %IA cm^−3^ for [^64^Cu]Cu-**8** and 4.64 ± 0.91 %IA cm^−3^ for [^64^Cu]Cu-**9** at 24 h, respectively. In the blocking group, the tumor uptake for [^64^Cu]Cu-**8** and [^64^Cu]Cu-**9** was 0.74 ± 0.24 %IA cm^−3^ and 2.16 ± 0.64 %IA cm^−3^ at 24 h p.i., respectively. Renal excretion was rapid with kidney uptake peaking at ~ 5 min p.i. for both compounds. After decreasing rapidly within the initial 20 min p.i., kidney accumulation for both radiotracers plateaued to ~ 3–5 %IA cm^−3^ at 1 h p.i. In contrast, a biphasic renal excretion was observed for the monomer and the trimer in the blocking group. Uptake in the liver was also rapid for both compounds peaking at 5 min p.i. and rapidly decreasing to a plateau of ~ 4–5 %IA cm^−3^ after 10 min p.i. No significant differences in liver uptake between the normal and blocking groups were noted for [^64^Cu]Cu-**8** and [^64^Cu]Cu-**9**.

Altogether, both compounds exhibited favorable pharmacokinetics with rapid and persistent accumulation in the U-87MG tumors together with rapid blood and kidney clearance suggesting that both radiotracers may find applications in PET imaging of α_v_ß_3_ integrin-positive tumors. Noteworthy, in spite of the 2.5-fold higher molecular weight of the trimer, the biodistribution profiles of both compounds were comparable but with a ~ 2.5-fold higher tumor uptake of the trimer, which may translate into higher sensitivity compared with the monomer.

## Conclusions

In this study, we developed the metal chelator NOTI-TVA **6** based on our chelating platform that allowed the covalent conjugation of three targeting vectors *via* peptide bond formation. A high yielding synthetic route for the NOTI-TVA scaffold was established allowing synthesis on a multigram scale. To investigate the feasibility of this trifunctionalized chelator for radiopharmaceutical development, biological evaluations in combination with three RGD targeting vectors were performed. Results suggest that aliphatic modifications at the non-coordinating NH groups of the imidazole residues are well tolerated and that these modifications had no measurable impact on the radiolabeling properties of the NOTI-TVA **6** scaffold with copper-64. This observation is in accordance with our previous findings on the non-conjugated metal chelators NOTI and NOTI-Me. Here, carboxylic acid groups were introduced for bioconjugation, but the system is highly flexible and can be readily adapted to other types for bioconjugation such as copper-free click chemistry. In line with reports on other multimeric RGD-based radiotracers, in cell experiments in the α_v_ß_3_-positive U-87MG cell line, the trimer displayed a higher binding affinity and internalization than its monomeric counterpart. The ^64^Cu-labeled trimer also displayed superior tumor uptake in *ex vivo* biodistribution and PET imaging studies with a ~ 2.5-fold higher tumor accumulation confirming the concept of increased local concentration at the target site by multimerization. Altogether, we showed that multiple functional groups for bioconjugation as well as additional peptidic targeting vectors can be introduced to NOTI **1** without compromising its metal binding properties. The straightforward modification of the imidazole arms of NOTI **1** by nucleophilic substitution paves the way for multiple applications of our chelating platform in radiopharmaceutical development. In combination with the recently described protection/deprotection strategy for the TACN macrocycle, not only compounds with up to three identical targeting vectors based on NOTI **1** are now at hand but, more importantly, also radiotracers comprising various different substituents such as different targeting vectors and/or fluorescent dyes are accessible by this approach.

## Electronic Supplementary Material

ESM 1 (PDF 1043 kb)

## Data Availability

Data sharing not applicable to this article as no datasets were generated or analyzed during the study.

## Abbreviations

BFC: bifunctional chelator
DIPEA: *N*,*N*-diisopropylethylamine
DMF: *N*,*N*-dimethylformamide
DOTA: 1,4,7,10-tetraazacyclododecane-1,4,7,10-tetraacetic acid
HATU: 1-[bis(dimethylamino)methylene]-1*H*-1,2,3-triazolo[4,5-b]pyridinium-3-oxide hexafluorophosphate
MIP: maximum intensity projection
NODIA-Me: 2-(4,7-bis((1-methyl-1H-imidazol-2-yl)methyl)-1,4,7-triazonan-1-yl)acetic acid
NOTA: 1,4,7-triazacyclononane-1,4,7-triacetic acid
PSMA: prostate-specific membrane antigen
RCP: radiochemical purity
RCY: radiochemical yield
TACN: 1,4,7-triazacyclononane
TFA: trifluoroacetic acid (TFA)
%IA g^−1^: percent of injected activity per gram
