# Supplementary material for: Proof-of-Concept Study of the NOTI Chelating Platform: Preclinical Evaluation of 64Cu-Labeled Mono- and Trimeric c(RGDfK) Conjugates
Source: Mol Imaging Biol. 2020 Aug 27;23(1):95–108. doi: 10.1007/s11307-020-01530-8 (PMC7782405; doi:10.1007/s11307-020-01530-8)
Supplement: Supplementary file 1 — (PDF 1043 kb) [file 11307_2020_1530_MOESM1_ESM.pdf]

Proof-of-concept study of the NOTI chelating platform: Preclinical evaluation of ^{64}Cu-labeled mono- and trimeric c(RGDfK) conjugates

SUPPORTING INFORMATION

Sebastian Martin[1,3], Stephan Maus[1], Tobias Stemler[1], Florian Rosar[1], Fadi Khreish[1], Jason P. Holland[4], Samer Ezziddin[1], and Mark D. Bartholomä[1,2]*

[1] Department of Nuclear Medicine, Saarland University – Medical Center, Kirrbergerstrasse, D-66421, Homburg, Germany

[2] Department of Nuclear Medicine, University of Freiburg – Medical Center, Hugstetterstrasse 55, D-79106, Freiburg, Germany

[3] Department of Nuclear Medicine and Molecular Imaging, Lausanne University Hospital, Rue de Bugnon 25A, CH-1011 Lausanne, Switzerland

[4] Department of Chemistry, University of Zurich, Winterthurerstrasse 190, CH-8057, Zurich, Switzerland

*** Corresponding Author:**
Dr. Mark D. Bartholomä
Tel: +49-(6841)-16-24664
Fax: +49-(6841)-16-24692
E-mail: mark.bartholomae@uks.eu

Characterization data compound 4

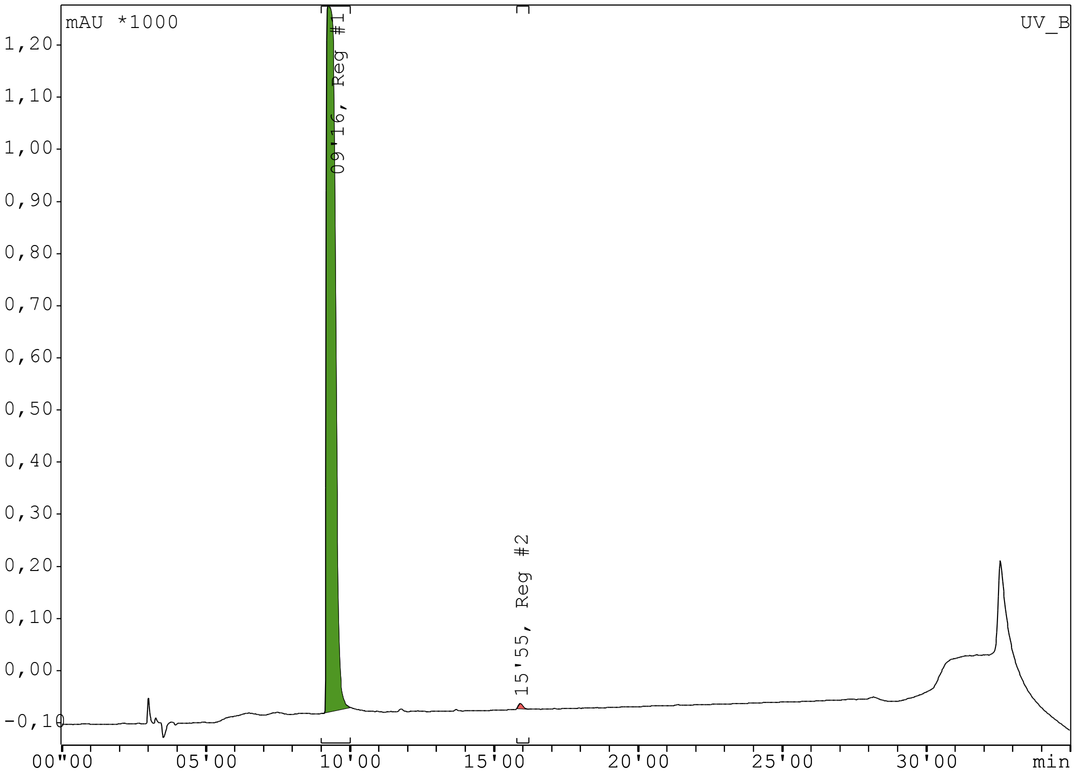

Figure S1. Representative analytical HPLC chromatogram of **4** (UV/vis trace).

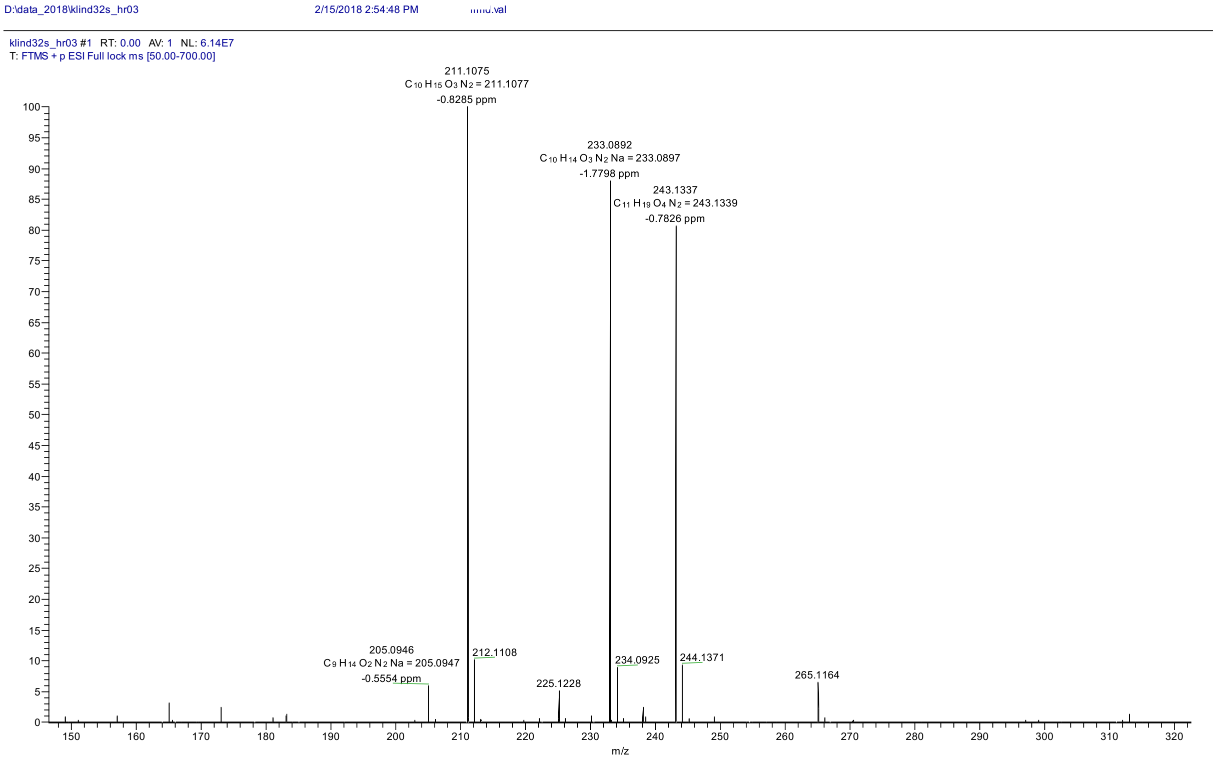

Figure S2. HR-ESI(+) spectrum of **4**.

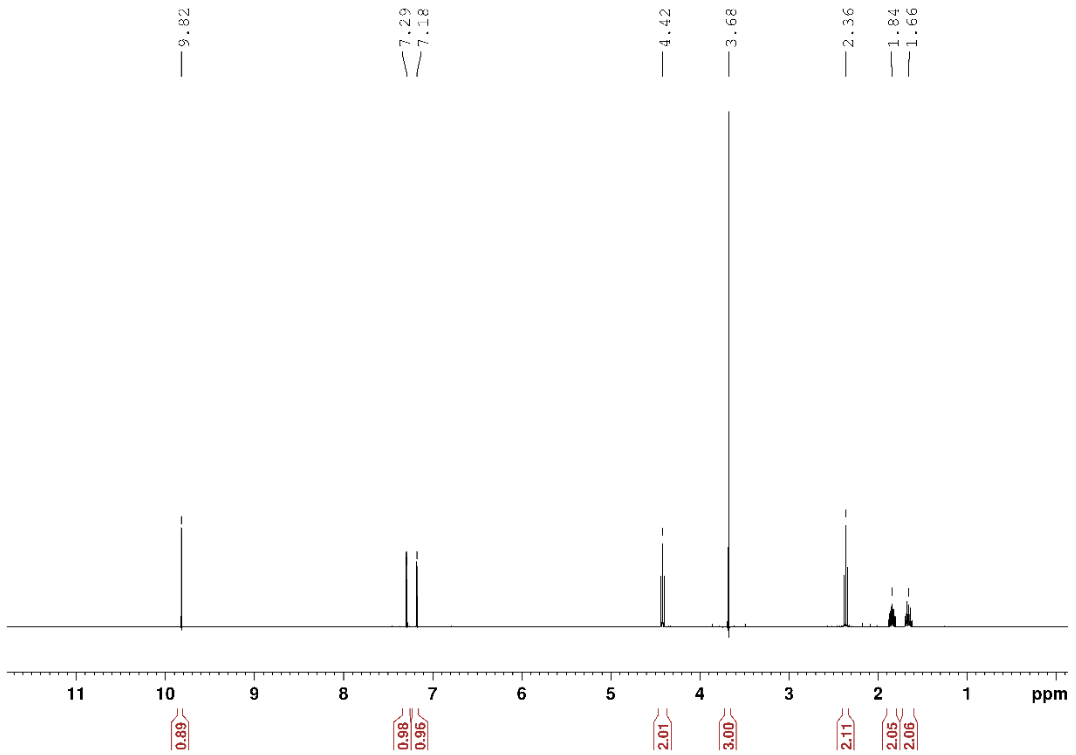

Figure S3. Proton NMR of **4**.

Characterization data compound 5

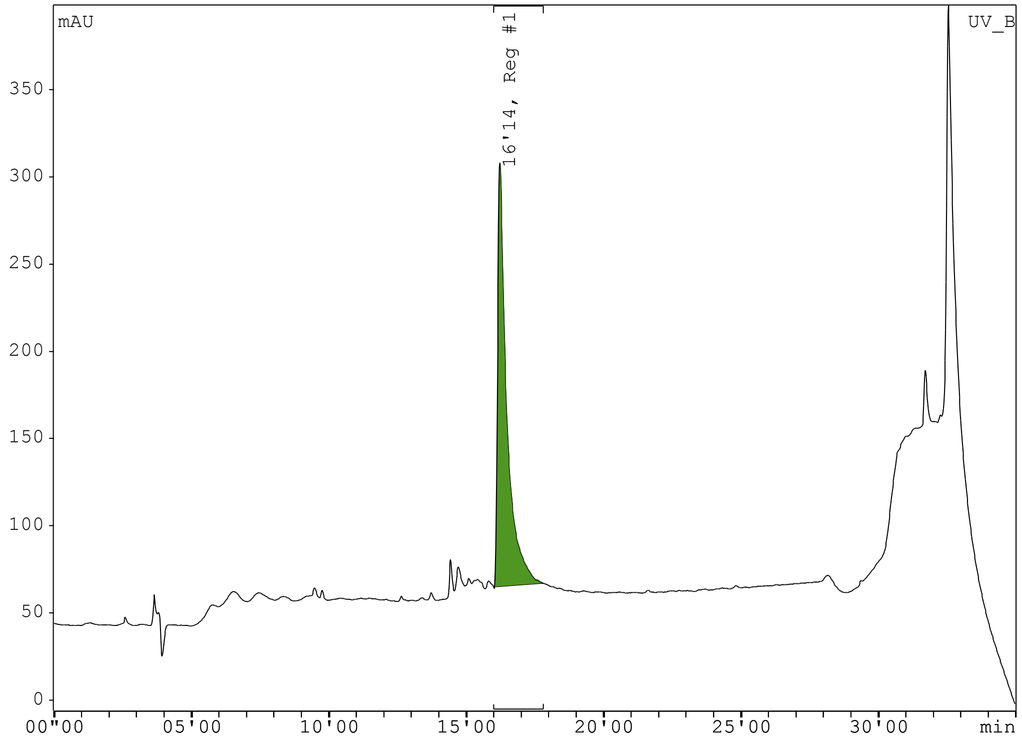

Figure S4. Representative analytical HPLC chromatogram of **5** (UV/vis trace).

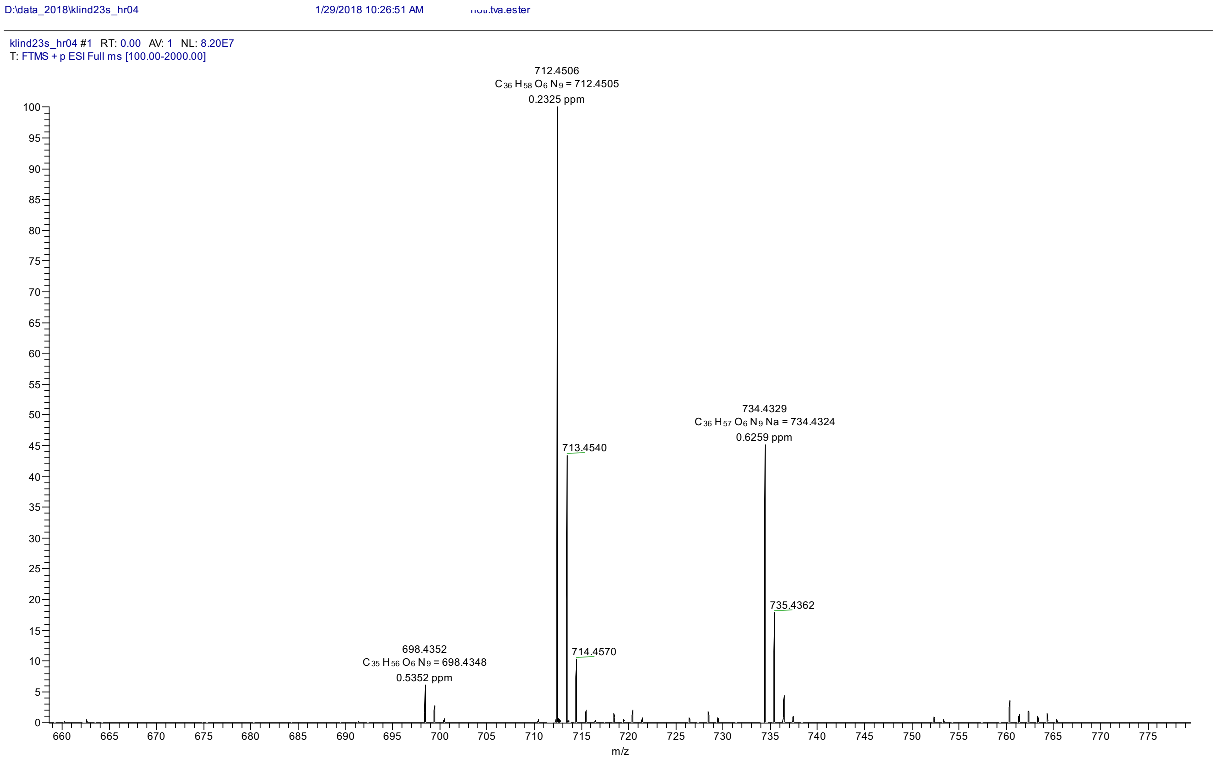

Figure S5. HR-ESI(+) spectrum of **5**.

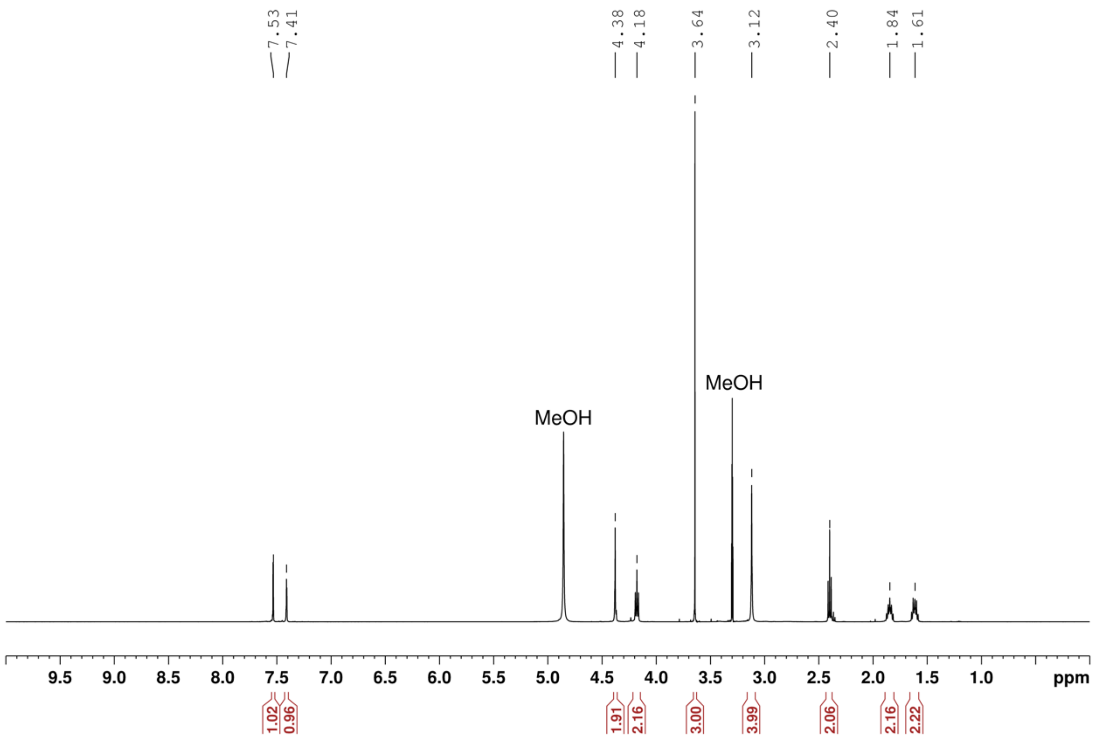

Figure S6. Proton NMR of **5**.

Characterization data compound 6

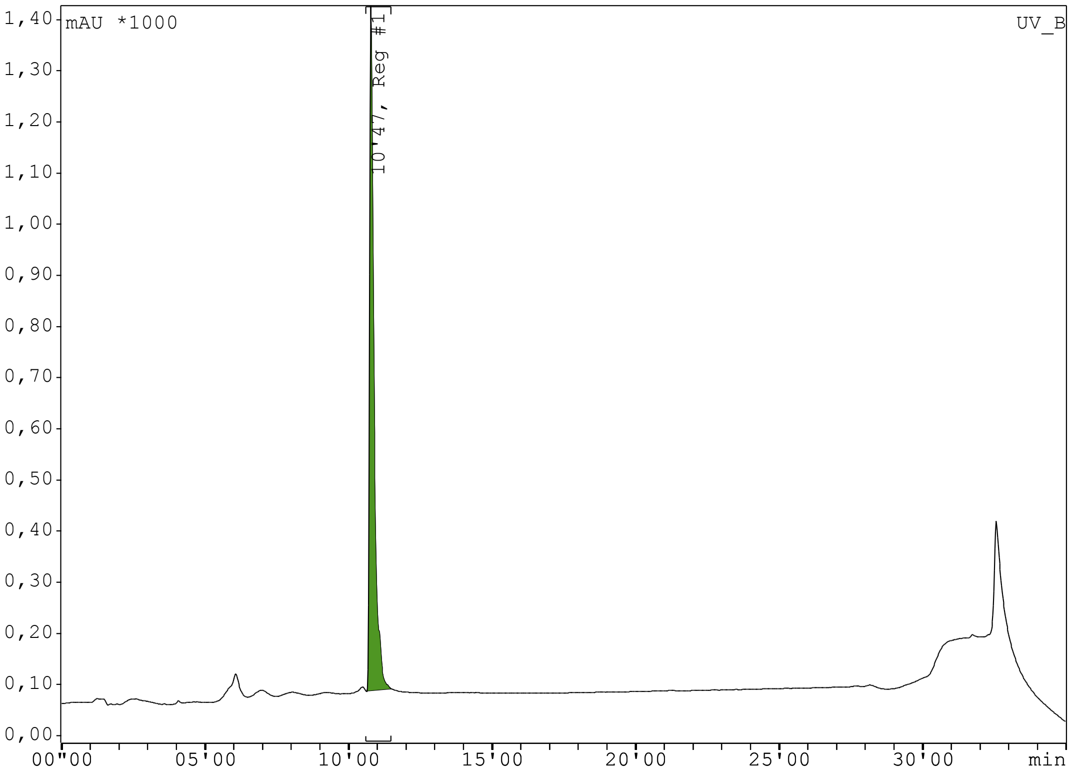

Figure S7. Representative analytical HPLC chromatogram of **6** (UV/vis trace).

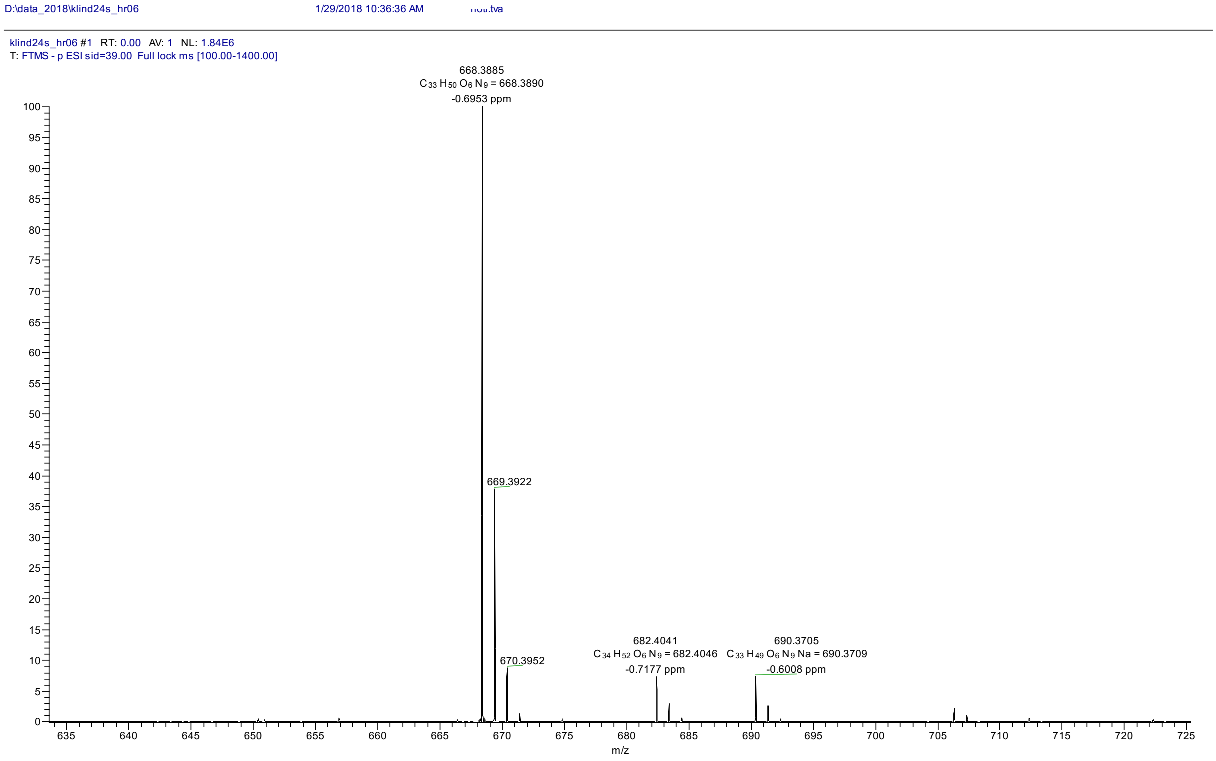

Figure S8. HR-ESI(+) spectrum of **6**.

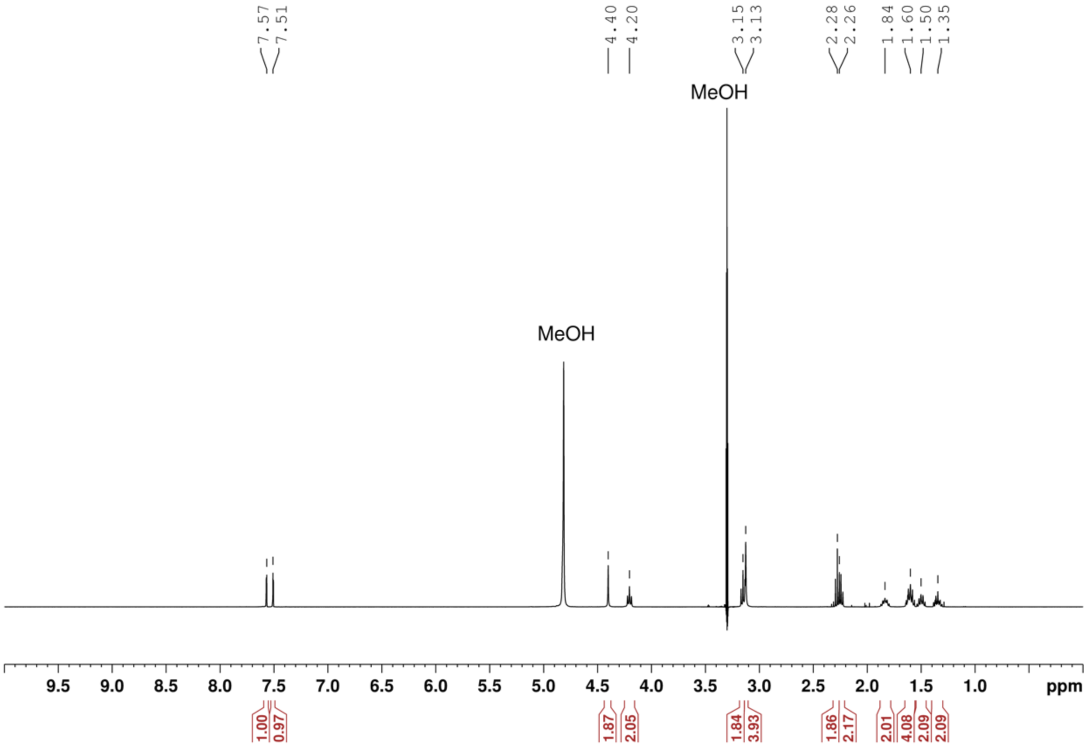

Figure S9. Proton NMR of **6**.

Characterization data of Cu-8/[^{64}Cu]Cu-8

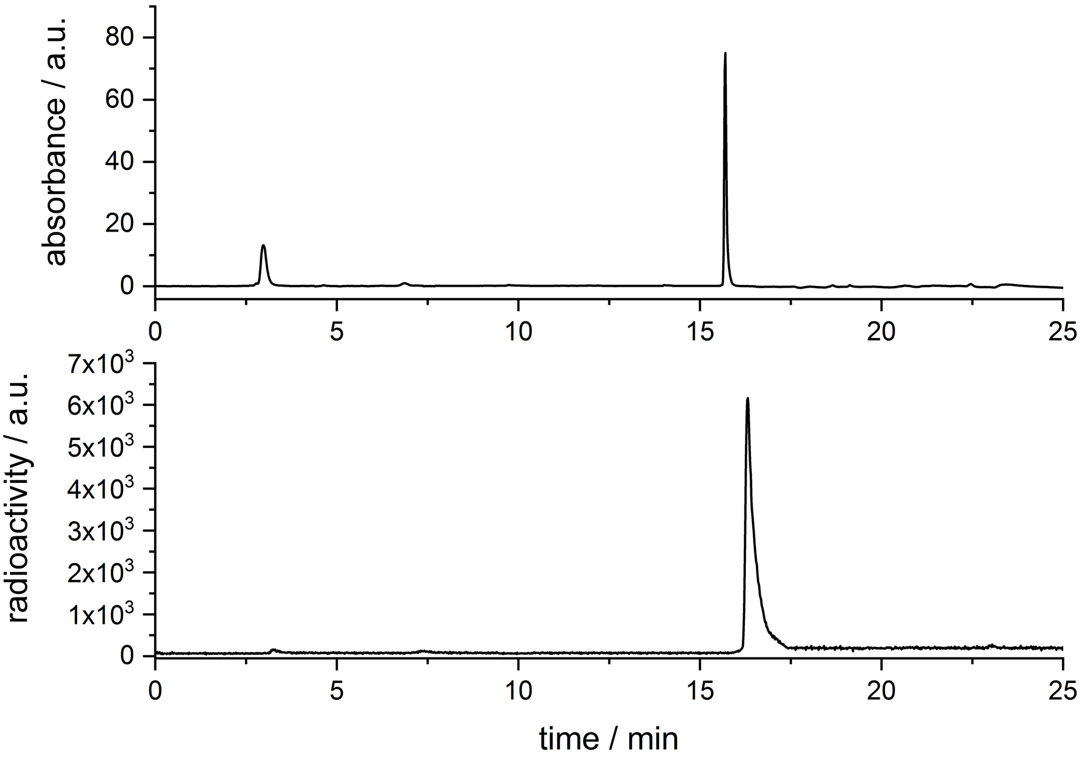

Figure S10. Representative analytical HPLC chromatograms of Cu-**8** (UV/vis; top) and [^{64}Cu]Cu-**8** (radioactivity; bottom).

Characterization data of Cu-9/[64Cu]Cu-9

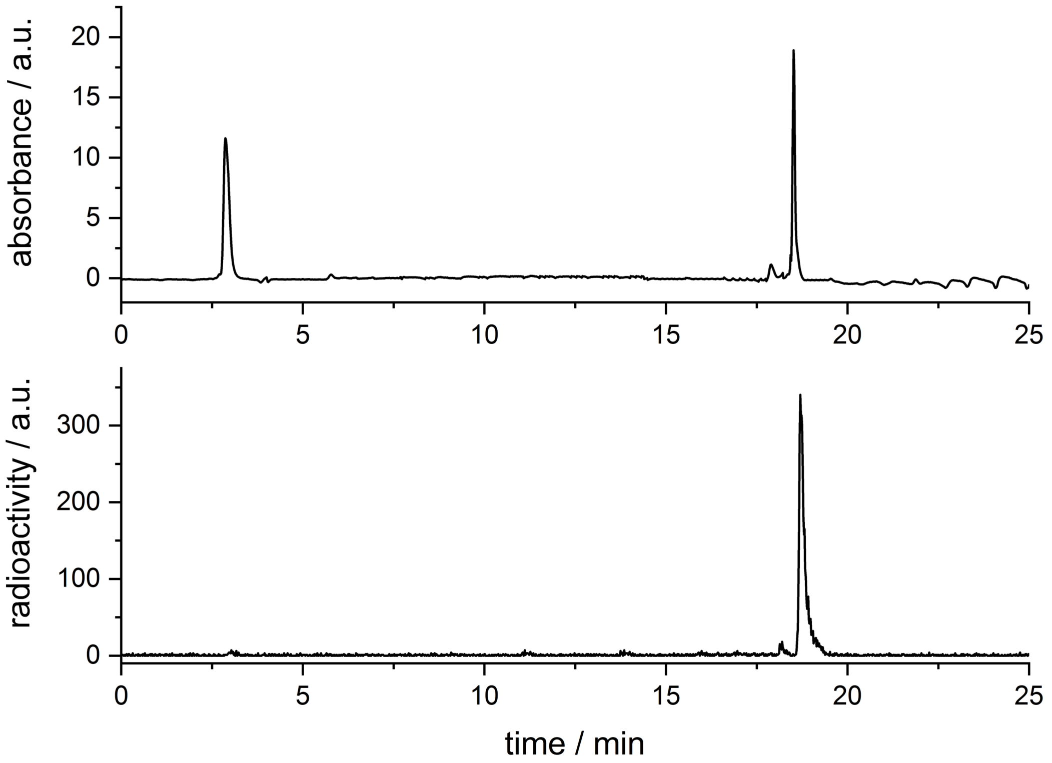

Figure S11. Representative analytical HPLC chromatograms of Cu-**9** (UV/vis; top) and [^{64}Cu]Cu-**9** (radioactivity; bottom).

Table S1. *Ex vivo* biodistribution of [^{64}Cu]Cu-**8** and [^{64}Cu]Cu-**9** in mice bearing $\alpha_v\beta_3$-positive U-87MG tumors at 1 h p.i. along with blocking studies. Data are expressed as %IA g^{-1} and represent mean ± SD (n = 3).

Organ	[^{64}Cu]Cu-**8**			[^{64}Cu]Cu-**9**		
	**1 h**	**1 h blockade**	***P* value***	**1 h**	**1 h blockade**	***P* value***
Blood	0.16 ± 0.05	0.05 ± 0.02	0.0199	0.34 ± 0.05	0.27 ± 0.09	0.3042
Heart	0.30 ± 0.02	0.06 ± 0.01	0.0001	1.03 ± 0.13	0.52 ± 0.07	0.0037
Lung	0.50 ± 0.34	0.14 ± 0.13	0.1653	2.81 ± 0.53	1.52 ± 0.23	0.0186
Spleen	0.72 ± 0.15	0.16 ± 0.01	0.0026	1.92 ± 0.24	0.65 ± 0.21	0.0011
Liver	2.75 ± 0.05	1.79 ± 0.26	0.0034	5.63 ± 1.61	3.32 ± 0.09	0.0675
Pancreas	0.30 ± 0.20	0.04 ± 0.01	0.0931	0.94 ± 0.37	0.46 ± 0.13	0.1038
Stomach	1.41 ± 0.43	0.13 ± 0.07	0.0068	1.66 ± 1.23	0.66 ± 0.37	0.2493
Intestine	0.73 ± 0.20	0.21 ± 0.05	0.0125	2.11 ± 1.15	1.73 ± 0.65	0.6462
Kidney	2.67 ± 0.33	1.59 ± 0.15	0.0070	7.65 ± 0.52	5.45 ± 0.59	0.0084
Muscle	0.28 ± 0.20	0.03 ± 0.01	0.0923	0.41 ± 0.06	0.29 ± 0.27	0.5046
Bone	0.35 ± 0.08	0.09 ± 0.03	0.0206	0.66 ± 0.23	0.38 ± 0.13	0.1289
Tumor	2.30 ± 0.28	0.26 ± 0.02	0.0002	5.57 ± 0.83	2.24 ± 0.11	0.0024
Tumor-to-blood	14.99 ± 3.05			16.22 ± 0.20		
Tumor-to-kidney	0.86 ± 0.04			0.73 ± 0.11		
Tumor-to-liver	0.83 ± 0.10			1.03 ± 0.26		
Tumor-to-muscle	10.53 ± 4.79			14.08 ± 3.83		

* statistical analysis of normal *vs.* blocking group using unpaired two-tailed t-test.
